# LINKING FAMILY TRANSITIONS AND LATER-LIFE DEPRESSION: DOES LIFE-COURSE SOCIOECONOMIC STANDING MATTER?

**DOI:** 10.1093/geroni/igz038.2137

**Published:** 2019-11-08

**Authors:** Claudia Recksiedler, Boris Cheval, Stefan Sieber, Robert S Stawski, Stephane Cullati

**Affiliations:** 1 German Youth Institute, Munich, Germany; 2 University of Geneva, Geneva, Switzerland; 3 Oregon State University, Corvallis, Oregon, United States

## Abstract

Research documented the impact of marital transitions—particularly marital loss—on depression in old age, yet its severity depends multiple factors. Individuals’ capability to cope with transitions depends on available resources and previous exposure to stressors, such as early-life adversity, which buffers or aggravates the impact of marital transitions on later-life depression. Although studies documented the pivotal link between early-life adversity and negative health trajectories, our study is the first attempt to examine whether early-life adversity influences the relationship between prospectively-tracked, later-life marital transitions and depression. We drew data from SHARE, which samples individuals aged 50+ across Europe (N = 13,258; 2004-2016). Using multilevel linear models, we found that women who became widowed had higher levels of depression compared to coupled and single women, but experienced lower increases in depression over time. After adjusting for early-life and adulthood SES, losing a partner remained significantly associated with depression. Life-course SES was associated with levels of depression, yet interactions between marital transitions and SES were not, with some exceptions: single women who reported difficulties in their ability to make ends meet experience higher increases of depression over time. Overall, results were similar for men. Interactions between family transitions and SES were again not significant, with a few exceptions for single men: those born in more childhood conditions, and those with high education, had lower levels of depression. We interpret and discuss our findings through the lens of life-course and stress-resiliency perspectives and in light of changing family dynamics for this age group.

